# Cholangioscope-Guided Electrohydraulic Lithotripsy as a Rescue Technique for an Impacted Dormia Basket With Large Common Bile Duct Stone

**DOI:** 10.14309/crj.0000000000000981

**Published:** 2023-02-22

**Authors:** Imran Ali Syed, Muhammad Farooq Hanif, Ahmad Karim Malik, Usman Iqbal Aujla

**Affiliations:** Gastroenterology and Hepatology Department, Pakistan Kidney and Liver Institute & Research Center, DHA Phase VI, Lahore, Pakistan

**Keywords:** Cholangioscope, Electrohydraulic Lithotripsy, Dormia Basket

## Abstract

Impaction of Dormia basket while extracting common bile duct (CBD) stones during endoscopic retrograde cholangiopancreatography is a well-known but relatively rare complication. Its management could be very challenging and may require percutaneous, endoscopic, or major surgical intervention. In this study, we present a case of a 65-year-old man with a history of obstructive jaundice secondary to a large CBD stone. For stone extraction, mechanical lithotripsy with a Dormia basket was attempted resulting in its entrapment within CBD. Subsequently, the entrapped basket and large stone were retrieved using a novel technique of cholangioscope-guided electrohydraulic lithotripsy with excellent clinical outcomes.

## INTRODUCTION

The estimated incidence of choledocholithiasis is 1%–15% in patients with cholelithiasis.^1^ Endoscopic retrograde pancreatocholangiography (ERCP) has replaced surgery as the primary treatment modality for the management of bile duct stones with success rates approaching 90%.^2^ Various ERCP-directed approaches have been defined in the literature to manage common bile duct (CBD) stones. The following table summarizes all such techniques (Table 1).

**Table 1.** Techniques to extract CBD stones^3,4^

A—By increasing biliary orifice using 1. Endoscopic sphincterotomy (ES). 2. Endoscopic papillary balloon dilation (EPBD). 3. Combination of ES and EPBD.
B—By reducing stone size (lithotripsy): 1. Mechanical lithotripsy. 2. Extracorporeal (shock wave) lithotripsy. 3. Intracorporeal (intraductal) lithotripsy using a cholangioscope (electrohydraulic, smart laser).

CBD, common bile duct.

Most stones can be extracted successfully using standard techniques, including endoscopic sphincterotomy, endoscopic papillary balloon dilation, or a combination of both, but some are more challenging because of their size, shape, and location and in a patient with surgically altered anatomy requiring more advanced endoscopic techniques.^5,6^

Mechanical lithotripsy is one of the established techniques for the extraction of challenging stones. It is not only cost-effective but also highly efficient with success rates ranging from 90% to 97%.^7^ A major complication while using a Dormia basket is its impaction either with an entrapped stone or due to malfunctioning of the traction wire during mechanical lithotripsy. The reported incidence varies between 0.8% and 6% in published series.^2,7–9^

In this report, we present a novel method of using cholangioscope-guided electrohydraulic lithotripsy (EHL) as a rescue technique for retrieval of an entrapped Dormia basket with an impacted large CBD stone.

## CASE REPORT

**Figure 1.**
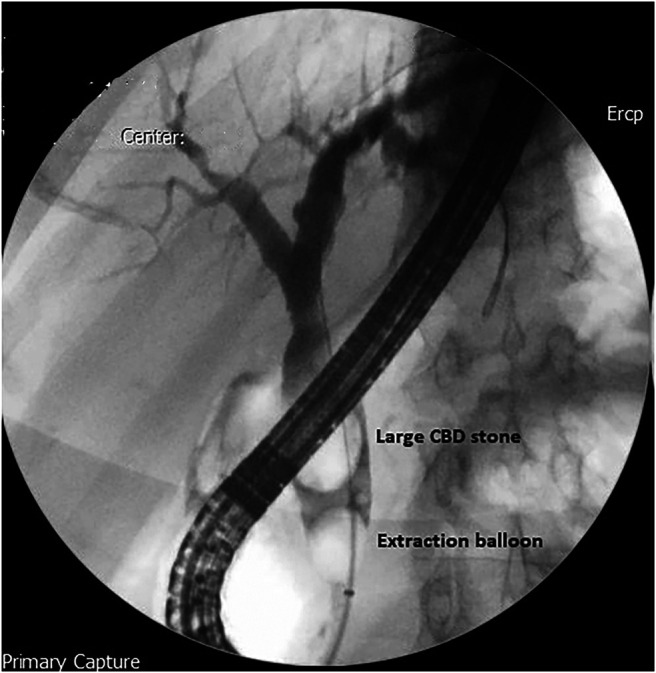
Cholangiogram showing an impacted large common bile duct stone.

A 65-year-old man presented with several weeks' history of intermittent jaundice with clay-colored stools, pruritus, and low-grade fever. He also reported a 6 kg weight loss during this period. His ultrasound scan revealed a contracted gallbladder with mild-to-moderate intrahepatic biliary dilatation and dilatated CBD measuring 24 mm with a large distally impacted CBD stone. Subsequently, an ERCP was performed, and a cholangiogram showed a large distal CBD stone of 20 mm with smooth tapering of distal CBD (Figure 1). Sphincterotomy was performed, and mechanical lithotripsy was attempted for stone extraction.

**Figure 2.**
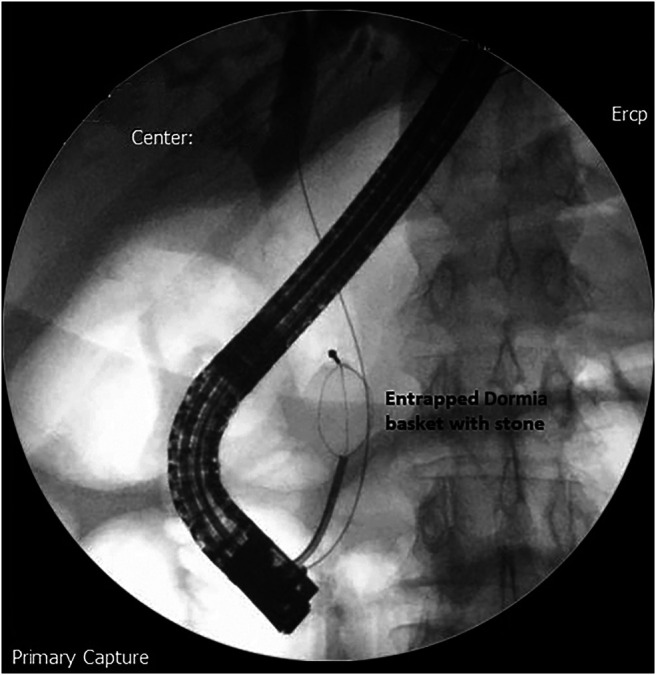
The impacted Dormia basket with a large CBD stone. CBD, common bile duct.

**Figure 3.**
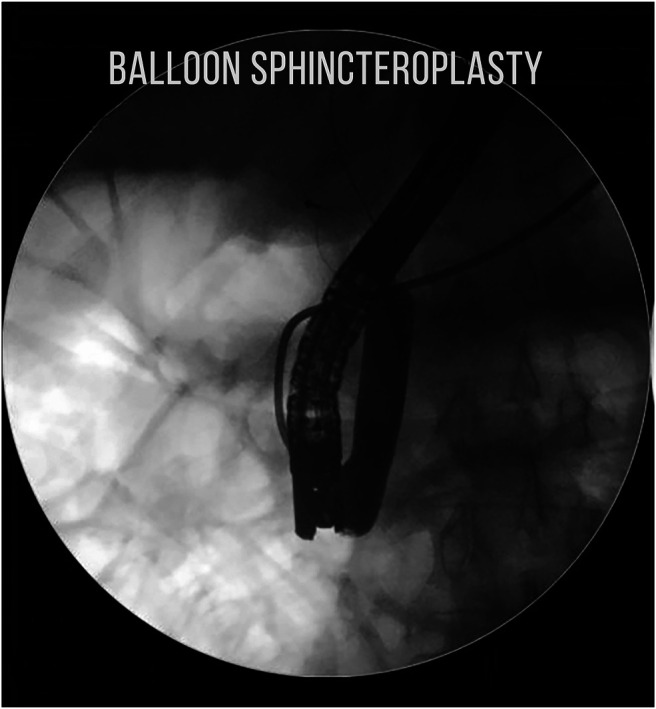
Balloon sphincteroplasty being performed after pushing the impacted Dormia basket and stone proximally.

**Figure 4.**
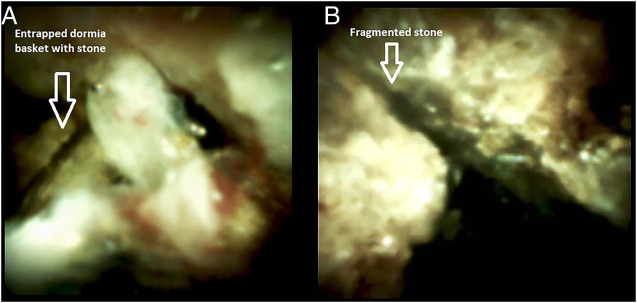
(A) Cholangioscope image showing wires of the entrapped Dormia basket. (B) Cholangioscope image showing the fragmented stone.

**Figure 5.**
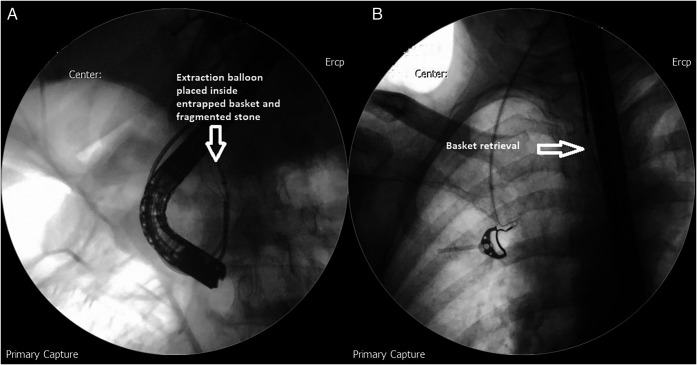
(A) Extraction balloon placed inside the entrapped Dormia basket and fragmented stone to dislodge the entrapped wires. (B) Retrieval of the Dormia basket.

**Figure 6.**
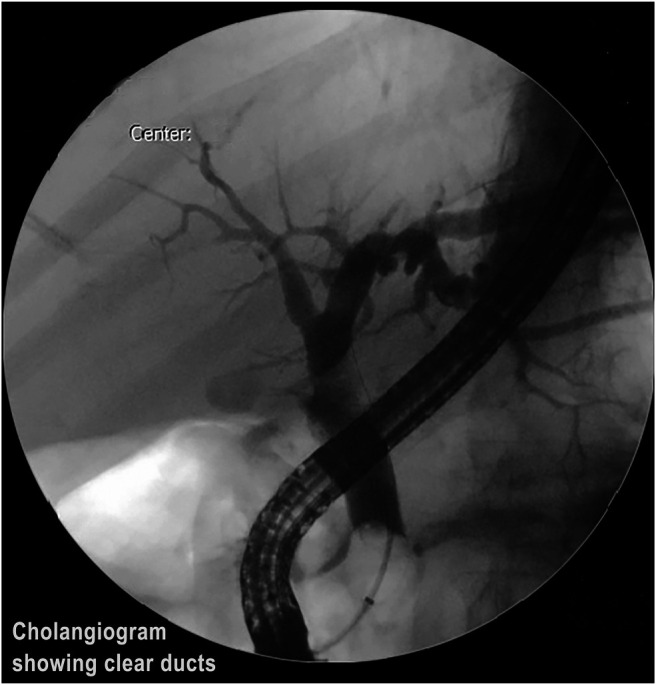
Occlusion cholangiogram showing clearance of bile ducts.

The stone was grasped with the Dormia basket, and multiple attempts to crush and retrieve it remained unsuccessful, resulting in the entrapment of the Dormia basket with the impacted stone (Figure 2). Balloon sphincteroplasty to 16 mm was performed for extraction of the entrapped basket and stone with no success (Figure 3). Subsequently, it was decided to use a novel technique of cholangioscope-guided EHL to dislodge and retrieve the entrapped basket. Basket wires were cut from the proximal end to release tension for the facilitation of retrieval. A single-operator intraductal cholangioscope was advanced with direct visualization of the stone and entrapped basket. EHL was performed for stone fragmentation (Figure 4). After fragmentation, a wire-guided extraction balloon was placed within the entrapped basket and fragmented stones. The extraction balloon was then inflated to 15 mm, which released the entrapped wires, and subsequently, the basket was retrieved successfully (Figure 5). Stone fragments were retrieved with the extraction balloon, and an occlusion cholangiogram confirmed clear ducts (Figure 6). The patient was discharged on the same day with no procedure-related complications.

## DISCUSSION

Choledocholithiasis is a common indication for ERCP. Large and difficult stones may become very challenging, even for an expert endoscopist. Mechanical lithotripsy is a commonly used technique for the extraction of complex CBD stones. However, it carries the risk of complications ranging from 3.6% to 35%, including entrapment of the Dormia basket around a large stone, fracture of Dormia basket wires, malfunction of the lithotripter handle, and damage to the CBD.^9,10^ Dormia basket impaction within CBD is one of the most challenging complications which may require endoscopic, percutaneous, and surgical interventions with related morbidity and mortality. Endoscopic techniques for the management of this complication include the extension of previous sphincterotomy, large balloon papillary dilatation, using a second basket (basketing a basket technique), extracorporeal shock wave lithotripsy, and endoscopic pulse-dye laser lithotripsy. Surgical techniques include laparoscopic retrieval or laparotomy and choledochotomy, whereas percutaneous approaches include transhepatic removal by the introduction of cholangioscope-guided EHL or by using a goose-neck snare.^7,11–13^ There is no consensus recommendation to prioritize rescue techniques for the retrieval of the entrapped basket, and it largely depends on individual expertise and available logistics. Surgery is the ultimate treatment option when all other approaches fail to retrieve the entrapped basket. In our case, this complication was managed successfully during the same procedure using a novel technique of cholangioscope-guided EHL with no procedure-related complications. This technique not only helped retrieve the entrapped basket and stone during the same endoscopic session but also avoided potential major surgery with excellent clinical outcomes. Before attempting cholangioscope-guided EHL, we tried to retrieve the entrapped basket with endoscopic papillary large balloon dilatation, but remained unsuccessful. This case emphasizes the effectiveness and safety profile of cholangioscope-guided EHL for the removal of the entrapped basket and stone. Nonavailability of cholangioscope-guided EHL in most general endoscopy units around the world is a major limitation, but referral networks and clinical pathways need to be established for the appropriate utilization of innovative techniques and resources to achieve better outcomes.

This case highlights the importance of the unique role of cholangioscope-directed EHL for the management of an impacted Dormia basket and should be considered as a preferred rescue modality when standard techniques remain unsuccessful in view of good clinical outcomes.

## DISCLOSURES

Author contributions: IA Syed wrote the manuscript and reviewed the literature. MF Hanif provided the cholangioscopic images and described the findings. AK Malik reviewed the literature. UI Aujla revised and edited the manuscript and is the article guarantor.

Financial disclosure: None to report.

Informed consent was obtained for this case report.
